# Approaches to Vaccination Among Populations in Areas of Conflict

**DOI:** 10.1093/infdis/jix175

**Published:** 2017-07-01

**Authors:** Chimeremma Nnadi, Andrew Etsano, Belinda Uba, Chima Ohuabunwo, Musa Melton, Gatei wa Nganda, Lisa Esapa, Omotayo Bolu, Frank Mahoney, John Vertefeuille, Eric Wiesen, Elias Durry

**Affiliations:** 1 Global Immunization Division, Centers for Disease Prevention and Control, Atlanta, Georgia; 2 Nigeria Polio Emergency Operations Center, National Primary Health Care Development Agency, Abuja, Nigeria; 3 National Stop Transmission of Polio Program, Africa Field Epidemiology and Training Network Nigeria Country Office, Abuja, Nigeria

**Keywords:** polio, immunization, vaccination, conflict settings, insecurity

## Abstract

Vaccination is an important and cost-effective disease prevention and control strategy. Despite progress in vaccine development and immunization delivery systems worldwide, populations in areas of conflict (hereafter, “conflict settings”) often have limited or no access to lifesaving vaccines, leaving them at increased risk for morbidity and mortality related to vaccine-preventable disease. Without developing and refining approaches to reach and vaccinate children and other vulnerable populations in conflict settings, outbreaks of vaccine-preventable disease in these settings may persist and spread across subnational and international borders. Understanding and refining current approaches to vaccinating populations in conflict and humanitarian emergency settings may save lives. Despite major setbacks, the Global Polio Eradication Initiative has made substantial progress in vaccinating millions of children worldwide, including those living in communities affected by conflicts and other humanitarian emergencies. In this article, we examine key strategic and operational tactics that have led to increased polio vaccination coverage among populations living in diverse conflict settings, including Nigeria, Somalia, and Pakistan, and how these could be applied to reach and vaccinate populations in other settings across the world.

Vaccination remains one of the most cost-effective disease prevention strategies, averting millions of childhood illnesses and deaths every year [1, 2]. Over the past 4 decades, advances in vaccine research and development, as well as improvements in vaccine delivery systems, have contributed to improved immunization coverage, resulting in historical declines in vaccine-preventable disease (VPD) morbidity and mortality worldwide [3, 4]. The eradication of smallpox in 1980 remains a remarkable testament to the effectiveness of mass vaccination campaigns as a disease prevention and control tool [5]. More recently, global efforts to eradicate wild poliovirus (WPV) have led to vaccination of millions of children and elimination of polio transmission in all but 3 countries [6, 7]. The impact of vaccination on other VPDs has also been remarkable. Endemic measles transmission has now been eliminated in the World Health Organization (WHO) Region of the Americas [8], and measles-related mortality has declined significantly across all continents [9]. An increasing number of countries have reported the elimination of maternal and neonatal tetanus, once considered a major cause of mortality, especially among new mothers and neonates [10]. With new funding available through major global initiatives, more countries continue to introduce new lifesaving vaccines into their national routine immunization schedules, while concurrently strengthening their immunization delivery systems, contributing to a trend of rising global immunization coverage rates [11].

While this remarkable progress in vaccine delivery continues, considerable gaps in accessing and immunizing children most in need of vaccines have become apparent, with evidence of the most acute gaps observed among children living in fragile nation states affected by conflicts. Outbreak of violence has been associated with dramatic reductions in immunization coverage. In the Syrian Arab Republic, the proportion of children vaccinated against 3 major VPDs—diphtheria, pertussis, and tetanus (DPT)—declined from preconflict levels of 80% in 2010 to 41% in 2015. In Ukraine, official WHO and United Nations Children’s Fund (UNICEF) surveys conducted in 2012 indicated that approximately 4 of 5 children were fully immunized with the DPT vaccine. Following 2 years of violent conflicts in parts of the country, however, in 2015, only 1 in 5 children surveyed were fully vaccinated with the DPT vaccine. Similar trends have been reported in other countries with a recent history of conflicts [12]. Globally, in 2015, of the estimated 20 million eligible infants who were not fully vaccinated with a DPT antigen, two thirds lived in countries affected by armed conflicts [13]. Additionally, UNICEF estimates that, in 2015, the 6 countries that reported third-dose coverage of <50% by the DPT vaccine—the Central African Republic, Equatorial Guinea, Somalia, South Sudan, Syrian Arab Republic, and Ukraine—were all experiencing conflicts or other humanitarian emergencies [14].

Populations in areas of conflict (hereafter, “conflict settings”) are at increased risk, particularly for polio and other VPD outbreaks, for a number of reasons. First, prevailing factors in these settings, including poor nutritional status, overcrowding, and unsanitary conditions, create a favorable milieu for VPD outbreaks. Second, the crippling of existing healthcare infrastructure due to armed conflict in these settings compromises immunization and other routine health services delivery systems, often creating a subpopulation of unimmunized or under-immunized persons susceptible to potential disease outbreaks. Third, when outbreaks occur in these settings, they spread rapidly, often with a high probability for prolonged transmission, owing to poor surveillance, poor or unavailable treatment facilities, poorly trained or unavailable health personnel [15], and difficulties associated with outbreak response planning and implementation. Finally, because of forced displacements across national and international boundaries, VPD outbreaks in these settings could result in large outbreaks elsewhere, often disseminated by clinically asymptomatic persons. All of these factors conspire to inflict excess disease burden and deaths on this vulnerable population and contribute to increased risk of disease spread worldwide [16].

Perhaps no other public health program more clearly illustrates the impact of armed conflicts on immunization access and utilization than the current Global Polio Eradication Initiative (GPEI). When the World Health Assembly resolved to eradicate polio in 1988, >350 000 polio cases were reported in 125 countries, all of which were then considered areas of polio endemicity [17]. Following 2.5 decades of successful polio immunization campaigns, polio has now been eliminated in most countries of the world, including countries with some of the weakest health-care delivery systems. In 2016, endemic polio remains confined to Afghanistan, Pakistan, and Nigeria, 3 countries that have experienced years of intractable armed conflicts [18]. Despite significant investments in eradication activities during the last 5 years, these 3 countries have been responsible for virtually all polio cases reported worldwide, including cases reported in previously polio-free countries. Furthermore, recent WPV and circulating vaccine-derived poliovirus reemergence in previously polio-free hot spots reflects a pattern of armed conflicts contributing to declining vaccination coverage, low population immunity, and reemergence of the poliovirus. In the Horn of Africa, widespread armed insurgencies in Somalia contributed to the significant increase in the population of unimmunized or underimmunized persons, leading to a large outbreak of poliovirus infection following importation of the virus in the country, which spilled over to neighboring Ethiopia and Kenya during 2013–2014 [19, 20]. During the same period, outbreak of armed conflicts in Syria and Iraq and the subsequent declines in immunization coverages in these countries were linked to the reemergence of WPV in the Middle East [21, 22]. Similar patterns have been observed with respect to the recent emergence of circulating vaccine-derived poliovirus infection outbreaks in many conflict settings in Nigeria, Cameroon, Chad, Niger, Myanmar, the Lao People’s Democratic Republic, and Ukraine [23].

Without developing and refining approaches to reach and vaccinate children and other vulnerable populations in conflict settings, polio and other VPDs may persist in these settings and spread across subnational and international borders. Implementing novel approaches to vaccinating children in conflict and humanitarian-emergency settings may save lives. In this article, using insights gained from the polio eradication program, we discuss strategic approaches and operational tactics that have been used to support polio vaccination amongst populations in a variety of conflict settings. Additionally, we discuss how these strategies could be applied in other settings to increase vaccination coverage for other VPDs.

## STRATEGIC APPROACHES TO VACCINATION IN CONFLICT AND HUMANITARIAN-EMERGENCY SETTINGS

A key feature of many 21st century armed conflicts is dispute over control of geographical territorial areas, typically by armed nonstate actors and putative government forces [15, 24]. These conflicts lead to varying degrees of insecurity, imposing limits to safe access to populations living behind conflict lines. While effective delivery of immunization services to populations in these settings may be fraught with varied levels of risk, experience in several geographical settings indicate that, with a good understanding of the nuances of the conflict and a great deal of operational flexibility, reaching and immunizing eligible populations in these settings may continue to be operationally viable. Key strategies that have been used in the polio eradication program include security assessments, negotiations with key actors, use of geographic information systems technology, close community engagement and coordination with other humanitarian relief activities, and flexibility around vaccine scheduling and dosing options.

### Security Assessments

Professional security assessments have become de rigueur in global public health because of rising levels of insecurity in several parts of the world. With these assessments, geographical locations are classified according to a risk continuum that is based on history of violence or other field intelligence assessments. Security assessments help immunization programs understand the risk profiles of areas where their work is performed, providing additional security guidance to health workers that is based on the most-recent intelligence assessments. In conflict settings, security assessments take on an added value and are core to planning and safe implementation of field activities. Regular revisions of security classifications is important, especially in settings with rapidly evolving security conditions. Incorporating local knowledge and information networks in security assessments is critical.

### Negotiating Secure Physical Access

Negotiating access to conflict areas, while complicated, may be key to effective delivery of vaccines and other health needs, as well as to conducting effective disease surveillance in these settings. While the nature of conflicts and motives of actors in various conflicts may vary, identifying and negotiating secure access with all conflict participants, including state and nonstate actors, as well as their allies, can be critical to minimizing field operational risks and may help improve the odds of effective vaccine delivery. Experience from the multidecade armed struggle in Colombia between the government and the Revolutionary Armed Forces of Columbia–People’s Army (commonly referred to as “FARC”) indicate that immunization coverage improvements even in rebel-held areas could be sustained through negotiation with key conflict actors. In other settings, including Somalia, putative governments and insurgent nonstate actors have allowed indirect access to populations by trusted individuals or organizations for purposes of providing immunization and other healthcare services in these settings. In Pakistan and Somalia, negotiated cessation of conflicts between armed parties allowed physical access to populations in conflict settings, with a number of vaccination rounds and polio surveillance conducted during the ensuing period [25, 26].

### Engaging Local Communities and Understanding Their Felt Needs

Communication and community engagement is a key element of successful vaccine delivery but is of critical value in vaccine delivery in conflict and humanitarian-emergency settings. Advocacy with local traditional and religious leaders, information sharing with communities, training of local residents as vaccinators, and building community mobilization networks with support from community “gatekeepers” may help shed light on the felt needs of the communities and build trust between the community and the program. Finding solutions to some of these needs, including working with other development partner agencies may be key to gaining trust, interest, and access to the community. In northern Nigeria, the Volunteer Community Mobilizer program, a focused initiative that recruited and trained local community women as social mobilizers and vaccination workers, is considered to have bolstered participation in house-to-house polio and other routine immunization programs, especially in security-compromised and hard-to-reach communities.

### Use of High-Resolution Geographic Information Systems Technology

In conflict settings, the use of high-resolution satellite imagery may provide program managers an insight into key population data that may guide vaccine delivery program planning and evaluation, including an estimate of target population and potential migration patterns. Recent high-resolution satellite images are now available for most of the world, and these can often be used to determine whether settlements have been damaged or abandoned or have a thriving population not otherwise accessible to vaccination. This tool is particularly important to guide decision-making in inaccessible areas. In Nigeria, the use of satellite imagery is currently being employed to develop a greater understanding of potentially eligible populations living in areas currently inaccessible to polio vaccination workers.

### Coordinating Vaccine and Other Humanitarian Aid Delivery

Because complex humanitarian emergency needs often coexist in many conflict settings, coordinating vaccine delivery efforts with other ongoing humanitarian response activities may be key to improving immunization coverage and efficient use of public health resources. In many settings, closely coordinating delivery of medicines, food, and clothing with vaccine delivery have helped improve vaccine reach in communities. In the Lake Chad region, efforts have been made to integrate nutritional assessments and interventions with polio vaccination.

### Flexible Age, Schedule, and Dosing Options

Insecurity compromises access to basic healthcare services and often means that multiple birth cohorts may have missed age-appropriate vaccines. Prioritizing flexibility around age and other eligibility criteria for receiving the vaccines is a key strategy for preventing outbreaks among populations living in conflict and humanitarian-emergency settings. These decisions are usually informed by epidemiological characterization of potential disease outbreaks and by the immunity profile of the population. In Nigeria and the Lao People’s Democratic Republic, catch-up campaigns have been used to vaccinate birth cohorts that missed recommended age-appropriate vaccines. Oral polio vaccine (OPV) has been administered among persons in all age groups instead of those in the typical 0–59 months age group. Concurrent administration of vaccine antigens, including those of the inactivated polio vaccine and OPV [27], which may have booster effects on polio immunity, have been conducted in conflict-affected Borno and Yobe states, Nigeria [28]. Additionally, in the face of potential supply shortages, the use of fractional-dose vaccines in these settings may be key to improving coverage.

## OPERATIONAL APPROACHES TO VACCINE DELIVERY IN CONFLICT SETTINGS

Depending on levels of access and prevailing risk assessments, a number of operational approaches may be available for safe delivery of vaccines to populations in conflict settings. Key strategies that have been used in the polio eradication program include opportunistic vaccination during days of tranquility, establishing barrier vaccination zones, collaborating with military and other security personnel, establishing permanent vaccination teams, strengthening vaccination activities at transit and border-crossing sites, and vaccinating at camps for refugee and internally displaced persons (IDPs) and other sites of mass gathering.

### Vaccination During Days of Tranquility

In certain conflict settings, negotiated or spontaneous lulls in violence may present windows of opportunity for vaccination [25]. This approach, sometimes referred to as “hit and run,” has been used during conflicts in the Democratic Republic of the Congo, Southern Sudan, Angola, Afghanistan, and Nigeria [29, 30]. This approach often requires rapid mobilization and deployment of substantial resources necessary to cover targeted areas during a relatively short period. A short-interval additional-dose strategy has been used to deliver vaccines in limited-accessibility areas.

### Barrier Vaccination Zones

Barrier vaccination zones may be established for containment of potential VPD transmission. Often referred to as “firewalling,” this strategy may involve setting up vaccination posts around areas inaccessible to vaccinators. Because populations in these areas may continue to move in and out of communities, vaccination posts are set up at security check points, border crossings, commercial bus and taxi stations, markets, and border settlement communities to reach populations traversing these areas. In addition to limiting potential spread of VPDs from the security-compromised areas, this approach may also help improve population immunity in the communities living in inaccessible areas. In Nigeria, firewalling has been implemented at all intrastate border crossings for Borno State, essentially forming a vaccine barrier around the state to stop potential spread of VPDs from inaccessible areas in the state to other parts of the country.

### Collaborating With Military or and Other Security Personnel

Military escorts have routinely been used in the delivery of humanitarian aid in conflict settings and may accompany vaccination teams during immunization exercises. In Pakistan, police and other civil security officers have served as escorts for vaccination teams [25]. In other settings, vaccination workers may embed with military contingents as they conduct routine security checks, providing an opportunity for vaccination during these missions. Members of medical corps can also be trained to administer vaccines and conduct disease surveillance. In Nigeria, volunteers within the civilian joint task force have been used to administer OPV during outbreak response vaccination activities in areas where civilian health workers cannot access.

### Permanent Vaccination Teams

Permanent vaccination teams are usually drawn from local communities and tasked with vaccinating eligible local populations. These vaccinators reside in the security-compromised areas and, as members of the local community, have the trust and access to local children that are otherwise not afforded to the staff of the formal vaccination program. Often set up as a discrete operation, these permanent vaccination teams are regularly supplied with vaccines and may receive supervision as security situation permits.

### Transit and Cross-Border Vaccination

Forced population displacement and increased migration are common features of conflict settings, providing an opportunity for vaccination at transit points such as bus stops and parking lots. Vaccinating migrating and refugee populations can also help limit VPD transmission and improve population immunity. In Nigeria and Pakistan, vaccination posts have been established at international border crossings. Customs officials in Nigeria have been trained and deployed for polio vaccination and AFP surveillance.

### Vaccinating at Refugee and Internally Displaced Person Camps and Other Mass Gathering Sites

Refugee and internally displaced person camps may provide an opportunity to access populations experiencing conflicts. Well-coordinated refugee camps have been used as a delivery point for both routine and supplemental immunization in conflict settings in many countries, including Kenya, Nigeria, and the Philippines [31]. Established camps will often have a clinic or other facility in which routine immunization services can be delivered. New or arriving refugees are screened for vaccination status, and catch-up vaccination may be provided. Religious institutions, including mosques, temples, churches, and other places of worship, and traditional or cultural gatherings have served as a useful access points to populations cut off from sites for vaccine delivery in conflict settings.

## DISCUSSION

Despite recent vaccine development advances and global immunization systems improvements, a substantial number of children in communities around the world remain partially or completely unimmunized, leaving them vulnerable to VPD morbidity and mortality. In resource-poor settings, poor vaccine access has historically been attributed to weak healthcare delivery infrastructure. However, recent GPEI experience increasingly highlights the role of armed conflicts as key drivers of poor immunization coverage, even in middle-income settings [4, 14]. Poor immunization coverages increase the risk of persistent VPD transmission, morbidity, and mortality [16].

We discussed key GPEI strategic and operational tactics that have contributed to increased polio vaccine delivery in conflict settings. Given the upward trend in the number of fragile nation states and the rising proportion of the global population projected to live in settings experiencing violent conflicts around the world [24], understanding and refining approaches to support continued vaccine delivery and disease surveillance in these settings may be of substantial value to the global VPDs control efforts.

Polio is now on the verge of eradication, aided in part by aggressive and innovative approaches to vaccine delivery worldwide, including in conflict and other humanitarian-emergency settings [17]. WPV transmission is now confined to Nigeria, Afghanistan, and Pakistan, all of which face ongoing conflicts [18]. In these settings, however, progress toward eradication continues to gather momentum, with an increasing number of children vaccinated and a trend of substantial declines observed in the number of cases reported annually. Additionally, when they have been reported, outbreaks of WPV and circulating vaccine-derived poliovirus infection have been successfully contained—and relatively quickly, through mass vaccination exercises, even in conflict and humanitarian-emergency settings [23]. This demonstrates that, with the right approaches, successful vaccine delivery that leads to the achievement of VPD elimination and eradication goals [11] may be attainable even in security-compromised areas.

While remarkable progress continues, considerable work remains to be done to achieve polio eradication worldwide. Accessing and immunizing all children in areas experiencing conflicts, especially in Nigeria, Afghanistan, and Pakistan, remains a major challenge to the polio eradication program. The risk of harm to vaccination teams remains a grave concern. Finding new methods and refining existing approaches to safely reach and vaccinate these populations should remain an important global public health priority.

In 2016, millions of children living in settings affected by armed conflict will face increased risks for disease and death because they were not reached by lifesaving vaccines. Applying lessons learned from the global polio eradication vaccination programs to reach and vaccinate these children may help reduce the number susceptible to VPD morbidity and mortality, saving lives worldwide.
